# Impact of Dietitian-Led Nutrition Therapy of Food Order on 5-Year Glycemic Control in Outpatients with Type 2 Diabetes at Primary Care Clinic: Retrospective Cohort Study

**DOI:** 10.3390/nu14142865

**Published:** 2022-07-13

**Authors:** Ayasa Nitta, Saeko Imai, Shizuo Kajiayama, Mikuko Matsuda, Takashi Miyawaki, Shinya Matsumoto, Shintaro Kajiyama, Yoshitaka Hashimoto, Neiko Ozasa, Michiaki Fukui

**Affiliations:** 1 Otsu City Hospital, Otsu 520-0804, Japan; ayasanman.n.vabo7915@gmail.com; 2 Department of Food and Nutrition, Faculty of Home Economics, Kyoto Women’s University, Kyoto 605-8501, Japan; takashiukb@gmail.com (T.M.); matumots@kyoto-wu.ac.jp (S.M.); 3 Kajiyama Clinic, Kyoto 600-8898, Japan; kajiyama-clinic@dream.ocn.ne.jp (S.K.); matsuda.mikuko@osaka-shoin.ac.jp (M.M.); kaji20091025abcd@gmail.com (S.K.); 4 Department of Endocrinology and Metabolism, Kyoto Prefectural University of Medicine, Graduate School of Medical Science, Kyoto 602-8566, Japan; y-hashi@koto.kpu-m.ac.jp (Y.H.); michiaki@koto.kpu-m.ac.jp (M.F.); 5 Department of Health and Nutrition, Faculty of Health and Nutrition, Osaka Shoin Women’s University, Osaka 577-8550, Japan; 6 Japan Red Cross Second Hospital, Kyoto 602-8026, Japan; 7 Department of Cardiovascular Medicine, Graduate School of Medicine, Kyoto University, Kyoto 606-8507, Japan; nei126@kuhp.kyoto-u.ac.jp

**Keywords:** type 2 diabetes, medical nutrition therapy: diet, dietitian, HbA1c, food order, long-term, primary care clinic, diabetic complications

## Abstract

The aim of this retrospective cohort study was to evaluate the effect of 5-year follow-up of dietitian-led medical nutrition therapy (eating vegetables before carbohydrates) on glycemic control in outpatients with type 2 diabetes (T2DM) at a primary care clinic. A total of 138 patients with dietitian-led medical nutrition therapy (intervention group) and 104 patients without dietitian-led nutrition therapy (control group) were compared for glycemic control, serum lipid, blood pressure, and diabetic complications for 5 years. Each patient in the intervention group received dietary education focused on food order (eating vegetables before carbohydrates) by dietitians. A significant improvement in HbA1c after 5 years in the intervention group [8.5 ± 1.7% (69 mmol/mol) to 7.6 ± 1.1% (59 mmol/mol), *p* < 0.001] was observed, whereas no change was observed in the control group [7.9 ± 1.2% (62 mmol/mol) to 8.0 ± 1.2% (63 mmol/mol)]. Dietary intake of protein, fat, carbohydrates, cholesterol, and salt in the intervention group demonstrated significant reduction, while the intake of dietary fiber significantly increased after the dietary education. Simple dietary education of ‘eating vegetables before carbohydrates’ presented by dietitians achieved good glycemic control after a 5-year period in outpatients with T2DM at primary care clinic.

## 1. Introduction

It is essential to manage good metabolic control in patients with diabetes in order to prevent chronic complications [1]. Meta-analyses have reported that the multidisciplinary approaches with the aim of making healthy changes in lifestyle can improve glycemic control and delay or reduce the progress of further complications by about 50–75% [2,3].

Patients with diabetes who were educated about comprehensive meal plans by registered dietitians have been shown to improve glycemic control and decrease prescribed medication [4]. However, a significant number of patients with diabetes with poor glycemic control remain, mainly as a result of low compliance and low adherence to medical nutrition therapy [5]. Medical nutrition therapy with daily food choices that affect blood glucose control in people with diabetes is conducted by patients themselves not by their physicians or dietitians. Therefore, nutritional education for patients has been found to benefit from patient-centered approaches encouraging self-management [6,7,8]. The medical nutrition therapy requires individual training by registered dietitians with counseling and coping skills and unequivocal information on evidence-based medicine [8]. Traditional dietary methods for diabetes in Japan mainly aim at consuming the appropriate diet, which focuses on restricting energy and carbohydrates and providing macronutrient balance [9]. However, even if patients understand the importance of medical nutrition therapy, quite a number of patients have difficulty keeping their appropriate daily food choices in real life over many years [5].

Registered dietitians have a national license and hospitals in Japan, but not necessarily primary care clinics, are required by law to hire registered dietitians. One of the roles of registered dietitians is to provide nutritional education to patients with diabetes, hypertension, hyperlipidemia, malnutrition, etc. The costs of medical nutrition therapy are covered by medical rewards provided by the Japanese government. However, some surveys report that about half of the patients with diabetes have not received diabetic-related knowledge and self-management skills at their primary care clinics and were not provided with the relevant educational programs [10]. One of the reasons for this is that registered dietitians are not required by law to be hired at primary care clinics in Japan.

We reported the significant acute and chronic effects of a simple and easy meal plan of ‘eating vegetables before carbohydrates’ by dietitians on the reduction of postprandial blood glucose concentration in outpatients with type 2 diabetes (T2DM) at primary care clinics [11,12], but long-term effects, i.e., more than 3 years, on glycemic control remain unknown. In this retrospective cohort study, we assessed the 5-year follow-up of dietitian-led individual medical nutrition therapy focused on food order of ‘eating vegetable before carbohydrates’ on glycemic control in outpatients with T2DM at primary care clinics.

## 2. Materials and Methods

### 2.1. Patients

We recruited diagnosed outpatients who fulfilled the World Health Organization (WHO) criteria for T2DM at the Kajiyama Clinic, which is the primary care clinic for diabetes in Kyoto, Japan. All patients who agreed to participate the study were informed of the importance of the dietitian-led medical nutrition therapy by the doctors at the clinic. The patients were divided into two groups as follows: patients who agreed to receive medical consultation by dietitians were included in the intervention group, while patients who refused to receive medical consultation by dietitians were included in the control group. The reasons for the patients rejecting the dietitian-led nutrition therapy were as follows: time limitation, economic burden, or the patients had already been educated in medical nutrition therapy by dietitians elsewhere. When the patients in the control group changed their mind and wanted to receive the dietitian-led nutrition therapy, they were included in the intervention group.

All participants received medical care from a physician every 1 to 2 months. The period of recruitment was from 2004 to 2009, and the 5-year follow-up was performed from 2004 to 2014. The exclusion criteria for participants were as follows: (1) chronic kidney disease stage higher than 4, (2) alcoholic or mental illness, (3) participants who had not received medical care at a clinic for more than 5 years.

The medical examination included body weight, systolic blood pressure (SBP), diastolic blood pressure (DBP), hemoglobin A1c (HbA1c), and serum concentration of total cholesterol (Total-C), low-density lipoprotein cholesterol (LDL-C), high-density lipoprotein cholesterol (HDL-C), and triglycerides (TG). The patients in the intervention group and the patients in the control group were compared in glycemic control, body weight, blood pressure, lipid profiles, and diabetic complications. Onset and progress of diabetic complications were assessed within and between the two groups retrospectively. Diabetic complications, including micro-vascular diseases (nephropathy, neuropathy) and macro-vascular complications (arteriosclerosis, coronary heart disease, cerebral vascular disease), were assessed based on clinical status. Diagnoses of nephropathy were based on the Classification of Diabetic Nephropathy [13] and Neuropathy on clinical examination findings, respectively [14]. The study was conducted in accordance with the Declaration of Helsinki. The study protocol was approved by the Ethics Committee of Kyoto Women’s University (27-10). Informed consent was obtained from all participants prior to the study.

### 2.2. Dietitian-Led Medical Nutrition Therapy of Food Order in the Intervention Group

A total 333 patients agreed to participate in the study, consisting of 196 patients in the intervention group and 137 patients in the control group at baseline (Supplementary Materials: Table S1). The patients in the intervention group received face-to-face medical nutrition therapy by dietitians every 1 to 2 months. The registered dietitians at the clinic were qualified in all aspects of instruction, counseling skills, and coping skills, which includes planning menus and making appropriate food choices.

**Figure 1:**
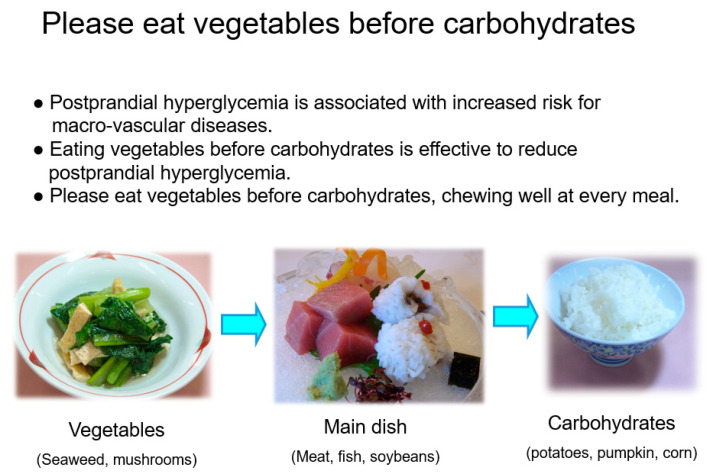
The original brochure for patients’ education, “Please eat vegetables before carbohydrates”.

In order to reduce postprandial glucose concentration, a simple and easy dietary method of ‘eating vegetable before carbohydrates’ was taught by the dietitians. We used the original brochure (Figure 1) and figure with the mean blood glucose concentrations to demonstrate the reduction in glucose excursions by eating vegetables before carbohydrates compared to the reverse regimen in both people with type 2 diabetes and with normal glucose tolerance [15]. This dietary strategy was focused on the food order of eating vegetables first for 5 min, then the main dish for 5 min, and then carbohydrates at the end for 5 min each meal [15]. The patients were recommended to eat vegetables, mushrooms, and seaweed in quantities of more than 120 g per meal in either raw or cooked forms and to decrease the intake of sweetened beverages, sweets, and fruits. Potatoes, pumpkin, and corn should be consumed last because they contain certain amounts of carbohydrates. The intervention focused on setting individual and realistic goals for glycemic control and achieving gradual dietary change according to the patient’s current dietary intake, dietary habits, and socioeconomic situations. The patients in the intervention group were interviewed by dietitians of the clinic who were able to retrieve more accurate dietary intake at the initial visit for pre-intervention to obtain the dietary intake at baseline. The patients in the intervention group reported 3-day dietary records after 3 to 6 months of medical nutrition therapy with the support of dietitians. Dietary intake was assessed and calculated for nutritional intake by the dietitians at the clinic using computer software (Eiyokun, Kenpakusya, Tokyo, Japan) to attenuate errors. Approximately 30 min were spent on the medical nutrition therapy at the initial visit and 20 min at subsequent sessions. On the other hand, the patients in the control group received routine doctor’s consultations every 1 to 2 months, and in the consultation, brief dietary advice was provided through the oral instruction “Please eat vegetables first”.

### 2.3. Laboratory Analyses

Laboratory data, body weight, and blood pressure were collected from all patients at baseline and every 1 to 2 months for 5 years at the clinic. Blood pressure was measured twice in the seated position during the physical examination after the patients had rested for 10 min. Blood samples were collected from all patients every 1 to 2 months at the clinic. HbA1c levels were determined by latex cohesion method (JCA-BM2250, KYOWA MEDEX, Co., Ltd., Tokyo, Japan); Total-C, TG, and LDL-C levels by enzymatic method (Bio Majesty JCA-BM 8060, JEOL, Ltd., Tokyo, Japa and HDL-C levels by direct method (Labospect 008K, Bio Majesty JCA-BM 8060, JEOL, Ltd., Tokyo, Japan).

### 2.4. Statistical Analysis

The primary outcome was HbA1c and the secondary outcomes were blood pressure and serum lipid concentrations. The results are expressed as mean ± SD unless otherwise stated. Baseline characteristics and the outcomes were compared between groups using chi-square, Mann–Whitney U test, or *t* test. Paired *t* test or Wilcoxon matched-pairs signed-rank test were used to analyze differences between baseline values and those after medical care. Differences were considered significant at *p* < 0.05. All analyses were performed with SPSS Statistics ver. 24 software (IBM Corp., Armonk, NY, USA).

## 3. Results

### 3.1. Characteristics of the Study Patients

**Figure 2:**
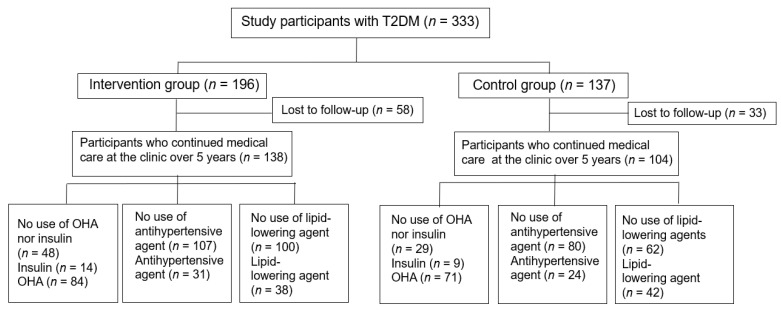
Study flowchart. T2DM, type 2 diabetes; OHA, oral hypoglycemic agents.

Excluding the patients who failed to keep the 5-year follow-up at the clinic, the results were based on 138 patients in the intervention group and 104 patients in the control group (Figure 2). In this study, 62 men and 76 women were included in the intervention group and 59 men and 45 women in the control group, with no significant differences in sex composition between the two groups. Since this was an observational study, the participants who agreed to receive medical consultation by dietitians were included in the intervention group, while the patients who refused to receive medical consultation by dietitians were included in the control group. There was no significant difference in age, sex, BMI, duration of diabetes, or blood pressure between the two groups at baseline except that HbA1c levels and LDL-C concentrations were significantly higher in the intervention group than those in the control group (Table 1). The mean number of individual dietary sessions was 11.2 ± 9.9 times (mean ± SD) and the mean duration of intervention was 11.4 ± 10.1 months in the intervention group.

### 3.2. Effects of Long-Term Glycemic Control, Blood Pressure, and Lipid Profile

The changes in BMI, HbA1c, blood pressure, and lipid profile for 5 years in both groups are shown in Table 2. Significant reductions in HbA1c were observed in the intervention group after dietary education during the 5-year period, whereas HbA1c in the control group did not show improvement during the 5 years. Comparing the two groups, the levels of HbA1c were significantly lower after dietary intervention in the intervention group than those in the control group for the 5-year period. Although significant reductions in SBP and DBP after medical treatment were demonstrated in both groups, after 5 years DBP was significantly lower in the intervention group than that of the control group. The significant reductions in Total-C and LDL-C concentrations after treatment in both groups were also demonstrated, but LDL-C concentrations in the control group after 5 years were significantly lower than those in the intervention group (Table 2).

In order to avoid the influence of the medication, we analyzed the patients not using insulin or oral hypoglycemic agents (OHA) after 5 years in both groups (Supplementary Materials: Table S2A). HbA1c was decreased significantly after 5 years in the intervention group [8.2% (66 mmol/mol) to 7.0% (52 mmol/mol)], whereas no change was observed in the control group [7.3% (56 mmol/mol) to 7.2% (55 mmol/mol)], although the baseline level of HbA1c was higher in the intervention group than that of the control group. In patients with untreated antihypertensive agents, both SBP and DBP demonstrated significant reductions after 5 years in the intervention group (SBP 127 to 124 mmHg, DBP 73 to 68 mmHg), while no changes were observed in the control group (SBP 129 to 128 mmHg, DBP 72 to 71 mmHg, Supplementary Materials: Table S2B). After 5 years, SBP and DBP in the intervention group were significantly lower than those in the control group. In patients not using lipid-lowering agents, Total-C concentration in the control group showed significant reduction after 5 years, although LDL-C, HDL-C, TG concentrations in both groups did not change after 5 years (Supplementary Materials: Table S2C). The number of patients using lipid-lowering medications significantly increased after 5 years in the control group, and the ratio of patients with lipid-lowering agents after 5 years was higher in the control group than in the intervention group (Supplementary Materials: Table S3).

Additionally, we analyzed the effect of sulfonylurea, which was used in more than half of the patients in both groups among the patients using OHA in this study. In the patients with sulfonylurea in the intervention group, HbA1c decreased after 5 years (8.5 ± 1.5% to 7.7 ± 1.4%, *p* < 0.01), whereas in the corresponding patients in the control group showed no difference in HbA1c after 5 years (7.5 ± 0.9% to 8.0 ± 1.1%, *p* = 0.567). Body weight in the intervention group (60.5 ± 12.0 kg to 57.9 ± 13.6 kg *p* = 0.773) and the control group (62.4 ± 10.5 kg to 62.1 ± 9.4 kg, *p* = 0.169) demonstrated no change during 5 years in patients with sulfonylurea. Additionally, the patients without use of sulfonylurea in the intervention group demonstrated reduction in HbA1c (7.9 ± 2.0% to 7.0 ± 0.9%, *p* < 0.01), whereas for the patients in the control group, HbA1c showed no difference before and after 5 years (8.1 ± 1.9% to 8.1 ± 1.3%, *p* = 0.530). As we observed in the patients with sulfonylurea, no change was observed in the body weight in both intervention group (64.1 ± 15.7 kg to 63.3 ± 13.3 kg, *p* = 0.230) and control group (59.9 ± 12.1 kg to 61.3 ± 13.6 kg, *p* = 0.530) of patients without use of sulfonylurea.

### 3.3. Changes in Nutritional Intake

The nutritional intake of the intervention group at baseline and after intervention is shown in Table 3A. The intake of energy, protein, fat, carbohydrates, cholesterol, and salt were all significantly decreased after the intervention in the intervention group, while intake of dietary fiber increased significantly. Among the food groups, while the intake of vegetables increased significantly, that of grain, meat, eggs, fruits, sweets, sweetened beverages, and oil decreased significantly after intervention. (Table 3B).

### 3.4. Changes in Diabetic Complications, Hypertension, Dyslipidemia, and Macro-Vascular Complications

Table 4 shows the change in micro-vascular (nephropathy and neuropathy) and macro-vascular complications (arteriosclerosis, cardiovascular disease, and cerebrovascular disease), hypertension, and dyslipidemia at baseline and after 5 years in both groups. The number of patients with diabetic complications in the intervention group showed no significant increase after 5 years except for diabetic neuropathy, but in the control group, the number of patients with diabetic neuropathy and arteriosclerosis significantly increased, and the ratio of patients with arteriosclerosis and dyslipidemia in the control group was higher than in the intervention group after 5 years.

## 4. Discussion

This retrospective cohort study demonstrated that this simple medical nutrition therapy focused on food order significantly improved the 5-year glycemic control in outpatients with T2DM at primary care clinics. To our knowledge, the current study is the first study to evaluate the long-term effect of dietitian-led medical nutrition therapy at primary care clinics in outpatients with T2DM. We observed a significant reduction in HbA1c in the intervention group after 5 years, and the impact of the effect on glycemic control remained significant in patients without using insulin or OHA. The intervention group showed a greater reduction in HbA1c by 0.9%, whereas the control group showed 0.1% increase, which achieved an overall difference of 1.0% after 5 years.

Furthermore, while the number of patients with diabetic complications in the intervention group showed no significant increase, in the control group the number of patients with dyslipidemia and arteriosclerosis increased, and the number of patients using lipid-lowering agent also increased significantly after 5 years.

As we have reported, the acute effect of this food-order regimen on reduction of postprandial blood glucose and the mean amplitude of glycemic excursions (MAGE) [11,15] might benefit the prevention of the long-term incidence of macro-vascular complications [16,17,18]. Additionally, decreased intake of meat and eggs and increased intake of vegetables and fish in patients in the intervention group led to the reduction of cholesterol intake, and it might be effective for preventing macro-vascular complications.

Large, randomized controlled studies have documented the effectiveness of nutrition interventions on the medical costs associated with diabetes [2,19]. The American Diabetes Association (ADA) reported that USD 37.3 billion in cardiovascular-related spending per year was associated with diabetes in the U.S.A. in 2017 [20]. The findings in the current study are important, namely that dietitian-led nutrition therapy at primary care clinics was successful in promoting patient education not only regarding 5-year glycemic control but also preventing macro-vascular complications. Therefore, the current nutrition therapy demonstrates the possibility of reducing long-term medical costs. Furthermore, most studies have been carried out with patients receiving medical care in academic hospitals or general hospitals with greater capacity to provide individualized nutrition counseling than that in primary care clinics [2,3,4]. There are several studies assessing the effect of dietitian-led nutrition therapy on glycemic control in patients with T2DM at primary care clinics [21,22], but in these studies the follow-up period was only 1-year, unlike the 5-year follow-up in the current study.

Patients with diabetes encounter several psychological and lifestyle difficulties when modifying their lives to achieve diabetic management, particularly when improving their diet according to medical nutrition therapy [23,24,25]. They often struggle with the restrictive behaviors of rigid daily nutrition therapy with regard to food choices and portions because they are often convinced that rigid nutrition therapy is the only way to obtain good glycemic control and avoid diabetic complications [5,9]. Therefore, the efforts of patients are difficult to keep in the appropriate direction for many years [5,23,24], and sometimes they receive confusing and contradictory advice from the media or social contacts.

The approach of eating vegetables before carbohydrates is easier to understand and to attain in making appropriate behavioral changes than other dietary approaches for patients with T2DM [11,12,15,26]. Since we published our studies showing the effect of food order (meal sequence) on glycemic control [11,12,15,26,27], the effect of food order has been discussed and confirmed internationally [28,29,30,31,32,33,34]. Particularly, this approach is also beneficial for medical professionals, especially for dietitians, because this nutrition therapy is easy to teach and takes only 20 min for each session. The mean duration of the individual dietary sessions by dietitians was about one year, so after the dietitian’s intervention the patients themselves could keep their glycemic control for another 4 years in the intervention group. Additionally, the dietitian-led medical nutrition therapy in the current study was effective when it was performed face to face, with a patient-centered attitude with each patient’s medical conditions, dietary habits, socioeconomic states, and psychological conditions, but the brief dietary education by doctors or nurses in the control group did not bring effective results. In the future, we need to establish a new method for other medical professionals to be engaged in food order nutrition therapy with the consideration of individual patients.

Our study showed important dietary behavioral changes in patients in the intervention group. The patients in the intervention group showed a significant increase in their consumption of vegetables and decreases in grains, sweets, and sweetened beverages after intervention. As a result, the intake of fiber increased, whereas the intake of carbohydrates and total energy decreased. Although this simple and easy nutrition therapy was not aimed at restricting energy and carbohydrate intake, the results showed the therapy was also effective in reducing the intake of energy and carbohydrates. The reason for the reduction in glycated hemoglobin levels in the intervention group can be explained by the decline in the intake of carbohydrates and the increase in the intake of dietary fiber in the vegetables consumed before the carbohydrates. [35,36,37,38,39]. It was shown that the diet of the intervention group lowered glycemic index by consuming vegetable before carbohydrates and that the carbohydrates consumed after vegetables and the main dish were digested slowly and required less insulin for subsequent metabolic responses [27,35,36,37,38,39,40]. This fact would be beneficial for Japanese patients with T2DM as it has been shown that the secretion of insulin in the Japanese is often delayed and that the ability for insulin secretion is about half that of Caucasians [41].

One of the reasons that the patients in the intervention group could keep their dietary plan long-term may be provided by the increased post-meal satiety and decreased subsequent hunger elicited by the increased consumption of vegetables [12,35,36,37,38,39,40]. This post-meal satiety may decrease the intake of carbohydrates, snacks, and sweetened beverages, with minimum physiological stress for patients in the intervention group.

Another interesting change was the decline in salt intake in the intervention group. The significant decrease in DBP after dietary intervention may be the result of decreased intake of salt. Because Japanese people usually consume boiled rice mixed with salted side dishes and main dishes [42], eating vegetable dishes first without mixing them with the boiled rice may have contributed to the reduction of salt, and it might have led to consequent improvement in blood pressure in the intervention group. On the other hand, no significant impact was shown in the lipid profile by the dietary intervention in the current study. However, further studies are required to determine the details of the mechanisms responsible for these metabolic changes.

There are some limitations which should be considered in this study. First, this study is a retrospective cohort study conducted at a single primary care clinic, and the number of dietary sessions and duration of intervention were not comparable among the patients. Additionally, because the patients in the control group refused to receive the dietitian-led nutrition therapy, the motivation for improving the dietary habits of the patients in the control group might have been insufficient compared to the intervention group. Additionally, the results were obtained from patients who continued receiving medical care at the clinic for 5 years in both groups, and thus, they may be considered as highly motivated patients and might have biased the results, although the ratio of the patients who continued receiving medical care for 5 years at the clinic was lower in the intervention group than that in the control group in the current study (70% in the intervention group vs. 76 % in the control group). Second, we could not compare the dietary intakes between the two groups because we intentionally did not request the dietary intake data from the patients in the control group. The reason for not requesting the data was that we considered that requesting the data itself may evoke consciousness in patients of their dietary habits, and we wanted to exclude the possibility that the consciousness in the patients in the control group may subsequently generate some kind of effect similar to those obtained in the intervention group. Additionally, the patients in the intervention group were interviewed by trained dietitians who were capable of retrieving more accurate dietary intake, which may attenuate errors, but some dietary records reported by patients might have been confounded by recall bias [43]. Third, we could not collect information on diabetic retinopathy despite it being one of the major micro-vascular complications. Fourth, since we did not measure serum insulin and incretin hormones in the patients, the present study could not analyze the relation between long-term glycemic control and hormone secretion and insulin resistance. Moreover, during the period from 2004 to 2009 in which the current study was started and conducted, sulfonylurea was used widely for patients with T2DM in Japan. Although new OHA, such as dipeptidyl peptidase 4 inhibitor (DPP-4 inhibitor) and sodium–glucose cotransporter 2 inhibitor (SGLT-2 inhibitor), are broadly used these days, they were not available for T2DM medication at that time. Therefore, the results of the current study should be translated carefully. 

Our results support the hypothesis that dietitian-led medical nutrition therapy focused on ‘eating vegetables before carbohydrates’ is effective for achieving good glycemic control and delaying further progress of diabetic complications for 5 years in outpatients with T2DM. This dietitian-led medical nutrition therapy of food order supports the concept of emphasizing how to eat, not just what to eat or how much to eat, with restrictions of energy and carbohydrate intake. Additional and longer studies are warranted to investigate the effects of this dietary approach for preventing the diabetic complications associated with T2DM and keeping good quality of life for patients. Thus, the dietitian-led nutrition therapy focused on eating vegetables first before carbohydrates approach may be a preferable approach as the first-line method in patients with T2DM.

## 5. Conclusions

The dietitian-led medical nutrition therapy of ‘eating vegetables before carbohydrates’ was effective in achieving good glycemic control over a 5-year period with setting individual and realistic goals for one’s glycemic control as well as introducing gradual dietary change according to the patient’s current dietary habits and socioeconomic situations in Japanese outpatients with T2DM at a primary care clinic. At each meal, the consuming order for food of eating vegetables first (more than 120 g per meal in either raw or cooked forms) followed by the main dish and carbohydrates last was recommended. Strategies for medical nutritional therapy should be re-organized to provide a simple and easy meal method focused on food order with ‘eating vegetables before carbohydrates’, expecting lower long-term glycemic control and the delayed onset and progress of diabetic complications on a lifelong basis in patients with T2DM.

## Figures and Tables

**Table 1:** Characteristics of patients with type 2 diabetes who continued to receive medical care at the clinic for 5 years at baseline for both groups.

	Intervention Group (*n* =138)	Control Group (*n* = 104)	*p*
Male/Female (*n*)	62/76	59/45	0.091
Age (years)	64.5 ± 11.0	66.4 ± 10.2	0.169
Duration of diabetes (years)	7.1 ± 8.2	7.8 ± 6.0	0.609
Body weight (kg)	61.3 ± 13.3	63.5 ± 12.5	0.184
BMI (kg/m^2^)	24.0 ± 4.4	24.5 ± 3.2	0.553
HbA1c (%) (mmol/mol)	8.5 ± 1.7 (69)	7.9 ± 1.2 (62)	0.005
SBP (mmHg)	132 ± 18	133 ± 15	0.786
DBP (mmHg)	75 ± 11	73 ± 10	0.307
Total-C (mg/dL)	213 ± 35	205 ± 34	0.086
LDL-C (mg/dL)	131 ± 30	123 ± 29	0.027
HDL-C (mg/dL)	56 ± 15	56 ± 15	0.668
TG (mg/dL)	140 ± 83	141 ± 77	0.898
Diet only			
No insulin or OHA, *n* (%)	48 (35)	29 (28)	0.361
No antihypertensive agent, *n* (%)	107 (78)	80 (77)	1.000
No lipid-lowering agent, *n* (%)	100 (72)	62 (60)	0.100
Prescribed medicine			
Insulin, *n* (%)	14 (10)	9 (9)	1.000
OHA, *n* (%)	84 (61)	71 (68)	0.375
Sulfonylurea, *n* (%)	61 (44)	62 (60)	0.033
Metformin, *n* (%)	18 (13)	31 (30)	0.005
α-GI inhibitor, *n* (%)	37 (27)	43 (41)	0.052
Glinide, *n* (%)	3 (2)	0 (0)	0.497
Thiazolidinedione, *n* (%)	8 (6)	7 (7)	1.000
Antihypertensive agent, *n* (%)	31 (22)	24 (23)	1.000
Lipid-lowering agent, *n* (%)	38 (31)	42 (42)	0.142

Data are mean ± SD or n. BMI, body mass index; SBP, systolic blood pressure; DBP, diastolic blood pressure; Total-C, total cholesterol; LDL-C, low-density lipoprotein cholesterol; HDL-C, high-density lipoprotein cholesterol; TG, triglycerides; OHA, oral hypoglycemic agents; α-GI inhibitor, alpha-glucosidase inhibitor.

**Table 2:** Changes in glycemic control, blood pressure, and lipid profile at baseline and after medical care in patients with type 2 diabetes for both groups.

	Intervention Group (*n* = 138)				Control Group (*n* = 104)			
	Baseline	After 1 Year	After 3 Years	After 5 Years	Baseline	After 1 Year	After 3 Years	After 5 Years
Body weight (kg)	61.3 ± 13.3	61.0 ± 12.6	61.6 ± 12.8	60.1 ± 14.3	63.5 ± 12.0	64.6 ± 11.1	64.5 ± 10.6	63.8 ± 13.3
BMI (kg/m^2^)	24.0 ± 4.4	23.8 ± 3.9	23.9 ± 3.9	23.8 ± 4.5	24.5 ± 3.2	24.5 ± 3.2	24.5 ± 3.0	24.4 ± 3.4
HbA1c (%) (mmol/mol)	8.5 ± 1.7 (69)	7.3 ± 1.0 (56) ***^†††^	7.5 ± 1.2(58) ***^†††^	7.6 ± 1.1 (59) ***^†^	7.9 ± 1.2 (62) ^††^	8.4 ± 1.1 (68) *	8.3 ± 1.0 (67) *	8.0 ± 1.2 (63)
SBP (mmHg)	132 ± 18	125 ± 13 ***	124 ± 9 ***	125 ± 11 ***	133 ± 15	126 ± 12 ***	127 ± 10 ***	128 ± 17
DBP (mmHg)	75 ± 11	71 ± 9 ***	71 ± 9 ***	68 ± 8 ***^†^	73 ± 10	70 ± 8 **	72 ± 7 *	71 ± 8
Total-C (mg/dL)	213 ± 35	210 ± 35	202 ± 33 **	198 ± 32 **	205 ± 34	199 ± 34 ^†^	197 ± 38 **	190 ± 31 ***
LDL-C (mg/dL)	131± 30	119 ± 28 ***	120 ± 30 **	120 ±30 *	123 ± 29 ^†^	119 ± 24	116 ± 26	110 ± 28 **^†^
HDL-C (mg/dL)	56 ± 15	57 ± 15	58 ± 16	56 ± 15	56 ± 15	56 ± 12	56 ± 15	57 ± 15
TG (mg/dL)	140 ± 83	131 ± 75	129 ± 70 *	139 ± 82	141 ± 77	140 ± 87	157 ± 117	139 ± 73

Data are mean ± SD. BMI, body mass index; SBP, systolic blood pressure; DBP, diastolic blood pressure; Total-C, total cholesterol; LDL-C, low-density lipoprotein cholesterol; HDL-C, high-density lipoprotein cholesterol; TG, triglyceride. Baseline vs. after intervention; * *p* < 0.05, ** *p* < 0.01, *** *p* < 0.001. Intervention vs. control group; ^†^ *p* < 0.05, ^††^ *p* < 0.01, ^†††^ *p* < 0.001.

**Table 3:** A. Changes in nutrient intake in patients with type 2 diabetes in the intervention group at baseline and after dietary intervention. B. Changes in intake of food groups at baseline and after dietary intervention in patients with type 2 diabetes in the intervention group.

	Baseline	After Intervention
A		
Energy (kcal)	2169 ± 91	1626 ± 46 ***
Protein (g)	76.8 ± 2.6	68.2 ± 2.0 **
Fat (g)	64.1 ± 3.7	45.4 ± 2.0 ***
Carbohydrates (g)	291 ± 14	221 ± 7 ***
Cholesterol (mg)	393 ± 43	257 ± 22 **
Dietary fiber (g)	13.7 ± 0.5	16.5 ± 0.7 **
Salt (g)	11.3 ± 0.3	9.1 ± 0.3 ***
B		
Grains (g)	459 ± 22	345 ± 16 ***
Potatoes (g)	40.3 ± 6.3	47.2 ± 7.3
Green vegetables (g)	91 ± 9	182 ± 15 ***
Other vegetables (g)	155 ± 11	221 ± 19 **
Mushrooms (g)	9.1 ± 2.2	12.1 ± 2.7
Seaweed (g)	5.4 ± 1.3	4.1 ± 0.9
Soy and soy products (g)	66.7 ± 9.4	68.9 ± 10.0
Fish (g)	88.6 ± 7.9	96.9 ± 8.2
Meats (g)	76.3 ± 8.7	61.2 ± 7.6 *
Eggs (g)	38.2 ± 3.9	27.6 ± 3.5 *
Daily products (g)	79.6 ± 12.3	85.0 ± 11.3
Fruits (g)	113.4 ± 15.6	67.3 ± 12.3 **
Sweetened beverages (g)	396 ± 44	321 ± 41 **
Sugar (g)	12.4 ± 2.1	6.9 ± 1.0 *
Sweets and snacks (g)	31.2 ± 4.9	17.6 ± 3.2 *
Nuts (g)	2.4 ± 0.7	2.0 ± 0.6
Oil (g)	16.8 ± 1.6	10.4 ± 1.1 **

Data are mean ± SE. Baseline vs. after intervention, * *p* < 0.05, ** *p* < 0.01, *** *p* < 0.001.

**Table 4:** Micro- and macro-vascular complications, hypertension, and dyslipidemia at baseline and after 5 years in patients with type 2 diabetes for both groups.

	Intervention Group (*n* =138)		Control Group (*n* = 104)	
	Baseline	After 5 Years	Baseline	After 5 Years
Diabetic nephropathy, *n* (%)	2 (1)	4 (3)	6 (6)	10 (10)
Diabetic neuropathy, *n* (%)	6 (4)	18 (13) *	4 (4)	17 (16) **
Arteriosclerosis, *n* (%)	56 (41)	59 (41)	39 (38)	64 (62) **^††^
Coronary heart disease, *n* (%)	12 (9)	14 (10)	10 (10)	17 (16)
Cerebrovascular disease, *n* (%)	16 (12)	21 (15)	12 (12)	23 (22)
Hypertension, *n* (%)	39 (28)	40 (29)	32 (31)	44 (42)
Dyslipidemia, *n* (%)	49 (36)	49 (36)	56 (54) ^†^	71 (68) ^†††^

Baseline vs. after intervention; * *p* < 0.05, ** *p* < 0.01. Intervention group vs. control group; ^†^ *p* < 0.05, ^††^ *p* < 0.01, ^†††^ *p* < 0.001.

## Data Availability

The data are not publicly available due to privacy reasons. Data supporting the reported results are available upon reasonable request and in accordance with the ethical principles.

## Supplementary Materials

The following are available online at https://www.mdpi.com/article/10.3390/nu14142865/s1, Table S1: Characteristics of participants with type 2 diabetes at baseline in both groups, Table S2A: Changes in glycemic control in patients with type 2 diabetes without use of insulin or OHA in both groups; Table S2B: Changes in blood pressure in patients with type 2 diabetes without use of antihypertensive agents in both groups; Table S2C: Changes in lipid profile in patients with type 2 diabetes without use of lipid-lowering agents in both groups; Table S3: The number of patients with type 2 diabetes with and without pharmacologic agents at baseline and after 5 years in both groups.
