# Supplementary material for: Impact of Dietitian-Led Nutrition Therapy of Food Order on 5-Year Glycemic Control in Outpatients with Type 2 Diabetes at Primary Care Clinic: Retrospective Cohort Study
Source: Nutrients. 2022 Jul 13;14(14):2865. doi: 10.3390/nu14142865 (PMC9322906; doi:10.3390/nu14142865)
Supplement: Supplementary file 1 [file nutrients-14-02865-s001.zip › nutrients-1771477-supplementary.pdf]

Supplementary Materials

Table S1. Characteristics of participants with type 2 diabetes at baseline in both groups

	Intervention group (*n* =196)	Control group (*n* = 137)	*p*
Male/Female (*n*)	99/ 97	62/ 75	0.345
Age (years)	62.8 ± 11.8	67.1 ± 9.3	0.000
Duration of diabetes (years)	7.1 ± 8.0	12.4 ± 9.2	0.000
BMI (kg/m^2)	24.2 ± 4.9	24.2 ± 4.0	0.758
HbA1c (%) (mmol/mol)	8.6 ± 1.8 (70)	8.2 ± 1.5 (66)	0.133
SBP (mmHg)	132 ± 17	138 ± 16	0.000
DBP (mmHg)	76 ± 11	75 ± 10	0.339
Total-C (mg/dL)	215 ± 37	210 ± 34	0.197
LDL-C (mg/dL)	131 ± 33	124 ± 29	0.059
HDL-C (mg/dL)	58 ± 16	57 ± 14	0.736
TG (mg/dL)	142 ± 83	140 ± 89	0.869
Diet only			
No insulin or OHA, *n* (%)	77 (39)	34 (25)	0.048
No antihypertensive agent, *n* (%)	153 (78)	99 (72)	0.414
No lipid-lowering agent, *n* (%)	132 (67)	70 (51)	0.031
Prescribe medicine			
Insulin, *n* (%)	30 (15)	36 (26)	0.079
OHA, *n* '(%)	110 (56)	87 (64)	0.312
Antihypertensive agent, *n* (%)	42 (21)	38 (28)	0.324
Lipid-lowering agent, *n* (%)	64 (33)	67 (49)	0.031

Data are mean ± SD or *n*. BMI; body mass index, SBP; systolic blood pressure, DBP; diastolic blood pressure, Total-C; total cholesterol, LDL-C; low density lipoprotein cholesterol, HDL-C; high density lipoprotein cholesterol, TG; triglyceride, OHA; oral hypoglycemic agents.

Table S2A. Changes in glycemic control in patients with type 2 diabetes without use of insulin or OHA in both groups

	Intervention group (n = 40)		Control group (n =23)	
	Baseline	After 5 years	Baseline	After 5 years
Male/female (n)	18/22	-	11/12	-
Age (years)	61.6 ± 10.6 †		69.1 ± 11.1	
Duration of diabetes (years)	2.6 ± 3.8 ††		9.0 ± 9.8	
BMI (kg/m^2)	24.5 ± 3.5	24.5 ± 3.7	25.4 ± 4.5	26.1 ± 2.8
HbA1c (%) (mmol/mol)	8.2 ± 2.0 (66)	7.0 ± 0.9 (52)**	7.3 ± 1.2 (56) †	7.2 ± 0.9 (55)

Data are mean ± SD or n. OHA; oral hypoglycemic agents, BMI; body mass index. Baseline vs. after intervention; $^{**}p < 0.01$, Intervention group vs. control group; $^{†}p < 0.05$, $^{††}p < 0.01$.

Table S2B. Changes in blood pressure in patients with type 2 diabetes without use of antihypertensive agents in both groups

	Intervention group (*n* = 95)		Control group (*n* =72)	
	Baseline	After 5 years	Baseline	After 5 years
Male/female (*n*)	41/54	-	47/25 ††	-
Age (years)	63.3 ± 11.0		66.0 ± 9.8	
Duration of diabetes (years)	5.6 ± 6.3		7.6 ± 6.1	
BMI (kg/m^2)	23.1 ± 3.3	23.0 ± 3.4	24.3 ± 2.8	24.6 ± 2.9
SBP (mmHg)	127 ± 13	124 ± 10 †	129 ± 14	128 ± 9
DBP (mmHg)	73 ± 10	68 ± 8 **†	72 ± 9	71 ± 8

Data are mean ± SD or *n*. BMI; body mass index, SBP; systolic blood pressure, DBP; diastolic blood pressure. Baseline vs. after intervention; $^{**}p < 0.01$. Intervention group vs. control group; $^{\dagger}p < 0.05$, $^{\dagger\dagger}p < 0.01$.

Table S2C. Changes in lipid profile in patients with type 2 diabetes without use of lipid-lowering agents in both groups

	Intervention group (n = 83)		Control group (n = 39)	
	Baseline	After 5 years	Baseline	After 5 years
Male/female (n)	32/51	-	24/15 †	-
Age (years)	62.7 ± 11.9		64.3 ± 10.4	
Duration of diabetes (years)	5.9 ± 7.4		7.1 ± 7.4	
BMI (kg/m^2)	23.9 ± 4.0	23.7 ± 3.8	25.3 ± 4.2	26.3 ± 3.8
Total-C (mg/dl)	209 ± 33	201 ± 33	200 ± 34	188 ± 30*
LDL-C (mg/dl)	129 ± 28	123 ± 28	121 ± 25	112 ± 29
HDL-C (mg/dl)	56 ± 16	55 ± 15	55 ± 14	55 ± 15
TG (mg/dl)	129 ± 76	141 ± 83	128 ± 50	134 ± 63

Data are mean ± SD or n. BMI; body mass index, Total-C; total cholesterol, LDL-C; low density lipoprotein cholesterol, HDL-C; high density lipoprotein cholesterol, TG; triglyceride. Baseline vs. after intervention; $^*p < 0.05$, Intervention group vs. control group; $^†p < 0.05$.

Table S3. The number of patients with type 2 diabetes with and without pharmacologic agents at baseline and after 5 years in both groups

	Intervention group (n = 138)		Control group (n = 104)	
	Baseline	After 5 years	Baseline	After 5 years
No use of insulin or OHA, n (%)	48 (35)	40 (29)	29 (28)	23 (22)
No use of antihypertensive agent, n (%)	107 (78)	95 (69)	80 (77)	72 (69)
No use of lipid-lowering agent, n (%)	100 (72)	83 (60)	62 (60)	39 (37) **††
Insulin, n (%)	14 (10)	14 (10)	9 (9)	12 (12)
OHA, n (%)	84 (61)	92 (67)	71 (68)	74 (71)
Sulfonylurea, n (%)	61 (44)	78 (57)	62 (60) †	65 (63)
Metformin, n (%)	18 (13)	36 (26) *	31 (30) ††	52 (50) **††
α-GI inhibitor, n (%)	37 (27)	39 (28)	43 (41)	48 (46) †
Glinide, n (%)	3 (2)	6 (4)	0 (0)	2 (2)
Thiazolidinedione, n (%)	8 (6)	9 (7)	7 (7)	14 (13)
Antihypertensive agent, n (%)	31 (22)	43 (31)	24 (23)	32 (31)
Lipid-lowering agent, n (%)	38 (28)	55 (40)	42 (40)	65 (63) **††

OHA; oral hypoglycemic agent. α-GI inhibitor; alpha-glucosidase inhibitor. Baseline vs. after 5 years; $^{*}p < 0.05$, $^{**}p < 0.01$, Intervention group vs. control group; $^{†}p < 0.05$, $^{††}p < 0.01$.
